# Associations between retinal thickness and background factors in eyes without retinal diseases

**DOI:** 10.1038/s41598-025-06863-4

**Published:** 2025-07-01

**Authors:** Yoko Ozawa, Noriko Onozato, Haruna Togawa, Shigeto Shimmura

**Affiliations:** 1 Department of Clinical Regenerative Medicine, Fujita Medical Innovation Center Tokyo; Eye Center, Fujita Health University, Haneda Clinic, Tokyo, Japan; 2 Department of Ophthalmology, Keio University School of Medicine, Tokyo, Japan

**Keywords:** Retina, Thickness, Sex, Blood test, Biomarkers, Medical research

## Abstract

Retinal thickness measured using optical coherence tomography (OCT) is a major parameter to evaluate retinal diseases. However, it may be influenced by systemic factors. We retrospectively analyzed OCT images and blood sample data from 266 participants (49.1 ± 10.5 years) including 181 (68.0%) males who underwent medical checkups at Fujita Medical Innovation Center, Tokyo. Those with retinal pathological findings were excluded. Males had thicker retinas in the center and inner circles of Early Treatment Diabetic Retinopathy Study grid (*P* < 0.01 for all). Mean thicknesses of the superior areas were greater than those of the inferior areas in inner and outer circles (*P* < 0.01 for both). However, there were eyes with thicker inner inferior areas (72 eyes, 27.1%), which was observed more frequently in males (*P* = 0.018). Thicker retinas were associated with lower hemoglobin A1c levels in the center (*P* = 0.012), and inner temporal (*P* = 0.042) and inferior (*P* = 0.047) areas; lower creatinine levels in the inner temporal (*P* = 0.002), superior (*P* = 0.026), and inferior (*P* < 0.001) areas; and higher high-density lipoprotein cholesterol levels in the inner nasal (*P* = 0.029) and inferior (*P* = 0.029) areas after adjusting for age and sex. These results may be kept in mind in evaluating OCT data during clinical practice and future clinical trials, although further studies are warranted.

**Supplementary Information:**

The online version contains supplementary material available at 10.1038/s41598-025-06863-4.

## Introduction

Recent progress in medical science has provided opportunities for clinical trials to develop new therapeutic approaches for neuroprotection. For retinal diseases, not only functional but structural findings recorded by multimodal imaging systems have become valuable: the image data were used as the primary outcome in the phase III clinical trials for developing new therapies for geographic atrophy related to age-related macular degeneration (AMD)^1,2^. In addition, retinal imaging systems are useful for exploring pathogenesis^3^. This emphasis on structural evaluation has promoted the study of retinal images in eyes with retinal diseases. However, the potential effects of background factors on the retina remain unclear.

The importance of the retinal structure in evaluating early and subtle changes has been well recognized in the field of glaucoma. Based on recent progress in optical coherence tomography (OCT), changes in the inner retinal thickness are involved in the definition of preperimetric glaucoma^4,5^. The sensitive detection of glaucomatous structural changes is a key to early diagnosis. Moreover, retinal structural changes, such as reduction in inner retinal volume in patients with diabetes without clinically diagnosed diabetic retinopathy, have been documented^6,7^. The fellow eyes of those with unilateral AMD, which have a high-risk for future AMD development^8–10^, exhibit shortening of the photoreceptor outer segments in OCT images^11^ that are most likely related to choriocapillaris flow deficits^12,13^.

Since the retina is composed of neural tissue that is nourished by circulatory flow, influences from the systemic background may affect retinal condition, not only those with retinal diseases. Thus, it is valuable to assess the association between retinal thickness and such demographics.

In this study, we analyzed retinal thickness measured using OCT and blood sample data obtained during medical checkups. Eyes with suspected retinal diseases and glaucoma were excluded. The potential influences of background factors were analyzed. In addition to sex differences, the associations between retinal thickness in each Early Treatment Diabetic Retinopathy Study (ETDRS) grid and blood sample data were analyzed. The information will help in understanding key points in evaluating OCT data during real-world clinical practice and in future clinical trials regarding neuroprotective therapies. It may also help in investigating the pathogenesis of retinal changes related to systemic conditions.

## Results

Mean age of the 266 participants was 49.1 ± 10.5 (range, 27–79) years, and 181 (68.0%) were males (Table 1). The mean retinal thicknesses in each ETDRS grid were: center, 237.8 ± 20.3 μm; inner temporal, 305.4 ± 14.5 μm; inner superior, 318.3 ± 15.2 μm; inner nasal, 316.8 ± 15.2 μm; inner inferior, 315.2 ± 14.4 μm; outer temporal, 264.9 ± 12.4 μm; outer superior, 276.1 ± 12.6 μm; outer nasal, 293.1 ± 15.0 μm; and outer inferior, 262.8 ± 13.1 μm (Table 1). The mean data from the blood tests were: hemoglobin A1c (HbA1c), 5.8 ± 0.6%; creatinine, 0.74 ± 0.19 mg/dL; total triglyceride (TG), 150.3 ± 108.3 mg/dL; total cholesterol (TC), 203.0 ± 41.8 mg/dL; high-density lipoprotein-cholesterol (HDLC), 56.6 ± 15.7 mg/dL; and low-density lipoprotein-cholesterol (LDLC), 121.8 ± 36.9 mg/dL (Table 1).

**Table 1:** Characteristics of the participants (*n* = 266).

Age	49.1 ± 10.5 [27–79, 48]
Sex (Male)	181 (68.0%)
Retina thickness	
Center (µm)	237.8 ± 20.3 [186.7-296.7, 238.0]
Inner Temporal (µm)	305.4 ± 14.5 [255.0-343.4, 305.4]
Inner Superior (µm)	318.3 ± 14.5 [269.6-355.9, 317.9]
Inner Nasal (µm)	316.8 ± 15.2 [263.4-357.8, 317.9]
Inner Inferior (µm)	315.2 ± 14.4 [268.8-350.6, 314.8]
Outer Temporal (µm)	264.9 ± 12.4 [227.6-299.3; 265.1]
Outer Superior (µm)	276.1 ± 12.6 [227.1-318.7; 276.5]
Outer Nasal (µm)	293.1 ± 15.0 [239.3-340.1; 293.2]
Outer Inferior (µm)	262.8 ± 13.1 [218.0-302.3; 263.7]
Systemic data	
Hemoglobin A1c (%)	5.8 ± 0.6 [4.5–9.8, 5.7]
Creatinine (mg/dL)	0.74 ± 0.19 [0.30–3.29, 0.73]
Triglycerides (mg/dL)	150.3 ± 108.3 [27–500, 115]
Total Cholesterol (mg/dL)	203.0 ± 41.8 [98–389, 203.5]
High-density lipoprotein cholesterol (mg/dL)	56.6 ± 15.7 [31–110, 53]
Low-density lipoprotein cholesterol (mg/dL)	121.8 ± 36.9 [20–319, 120]

Data are presented as mean ± standard deviation [range, median]. Retinal thicknesses of the right eyes were analyzed using the Early Treatment Diabetic Retinopathy Study grid chart (center, inner, and outer circles) in optical coherence tomography images.

Males had significantly thicker retinas in the center, all areas of the inner circles (*P* < 0.01 all), and temporal area of the outer circle (*P* = 0.031) (Table 2). The thicknesses in the other areas of the outer circles did not differ between the sexes. Analyses between the age groups divided according to the median value showed that younger group had thicker retina in the outer superior area (*P* = 0.049), although there were no significant differences in the other areas (Supplementary Table 1).

**Table 2:** Differences in retinal thickness between males and females.

Factors	Males (*n* = 181)	Females (*n* = 85)	*P* value
Age	48.7 ± 10.5	49.7 ± 10.5	0.447
Retina thickness			
Center (µm)	240.3 ± 20.4	232.4 ± 19.0	0.002**
Inner Temporal (µm)	307.7 ± 14.5	300.6 ± 13.3	< 0.001**
Inner Superior (µm)	319.9 ± 15.0	314.8 ± 12.8	0.007**
Inner Nasal (µm)	319.2 ± 15.0	311.7 ± 14.4	< 0.001**
Inner Inferior (µm)	317.2 ± 14.6	311.0 ± 13.0	< 0.001**
Outer Temporal (µm)	266.0 ± 12.8	262.4 ± 11.0	0.031*
Outer Superior (µm)	275.7 ± 13.5	277.0 ± 10.7	0.410
Outer Nasal (µm)	293.0 ± 15.7	293.0 ± 13.4	0.914
Outer Inferior (µm)	262.5 ± 13.4	263.5 ± 12.5	0.753

Data are presented as mean ± standard deviation. Retinal thicknesses were analyzed using the Early Treatment Diabetic Retinopathy Study grid. Mann-Whitney U test. *P < 0.05, **P < 0.01.

The retinal thicknesses in each area of the ETDRS grid in the participants were correlated (Table 3, *P* = 0.012 between the center and outer temporal areas and *P* < 0.01 for the others), except for the retinal thicknesses of the center and outer inferior areas, although there was a trend (*P* = 0.072). Then, the retinal thicknesses of the superior and inferior areas of the inner and outer circles were compared (Table 4). The mean superior retinal thickness was greater than the mean inferior retinal thickness in the inner and outer circles (both *P* < 0.001). Superior retinal thickness was thicker than that in inferior retina in most eyes; however, a thicker retina in the inferior area occurred in 72 eyes (27.1%) regarding inner circle and 8 eyes (3.0%) regarding outer circle. In particular, a thicker inner inferior retina was more frequently observed in males than in females (*P* = 0.018).

**Table 3:** Correlations of the retinal thickness between the ETDRS grid areas.

		Center	Inner Temporal	Inner Superior	Inner Nasal	Inner Inferior	Outer Temporal	Outer Superior	Outer Nasal
Inner Temporal	R	0.581							
	P	< 0.001**							
Inner Superior	R	0.520	0.919						
	P	< 0.001**	< 0.001**						
Inner Nasal	R	0.652	0.893	0.913					
	P	< 0.001**	< 0.001**	< 0.001**					
Inner Inferior	R	0.535	0.919	0.912	0.916				
	P	< 0.001**	< 0.001**	< 0.001**	< 0.001**				
Outer Temporal	R	0.155	0.625	0.663	0.564	0.668			
	P	0.012*	< 0.001**	< 0.001**	< 0.001**	< 0.001**			
Outer Superior	R	0.187	0.575	0.726	0.599	0.658	0.815		
	P	0.002**	< 0.001**	< 0.001**	< 0.001**	< 0.001**	< 0.001**		
Outer Nasal	R	0.198	0.576	0.708	0.633	0.688	0.681	0.846	
	P	0.001**	< 0.001**	< 0.001**	< 0.001**	< 0.001**	< 0.001**	< 0.001**	
Outer Inferior	R	0.111	0.520	0.606	0.527	0.644	0.789	0.840	0.848
	P	0.072	< 0.001**	< 0.001**	< 0.001**	< 0.001**	< 0.001**	< 0.001**	< 0.001**

Pearson correlation coefficient. R, correlation coefficient. ETDRS grid, Early Treatment Diabetic Retinopathy Study grid. *P < 0.05, **P < 0.01.

**Table 4:** Differences in retinal thickness between superior and inferior areas of the ETDRS grid.

ETDRS grid	*P* ^a^	Superior < Inferior (eyes [%])		*P* ^b^
		Total	Males	
Inner circle	< 0.001**	72 [27.1]	57 [79.2]	0.018*
Outer circle	< 0.001**	8 [3.0]	7 [87.5]	0.231

^a^Paired t-test to evaluate the difference of mean value. ^b^Chi square test to evaluate the number of eyes in males which showed thicker retinal thickness in inferior parts. ETDRS grid, Early Treatment Diabetic Retinopathy Study grid. *P < 0.05, **P < 0.01.

The associations between retinal thickness in each area and blood data were analyzed after adjusting for age and sex (Table 5). The eyes with thicker retina than the median value were compared with those without in each ETDRS grid area. The HbA1c levels were negatively associated with thicker retinas in the center (*P* = 0.012), inner temporal (*P* = 0.042), and inner inferior (*P* = 0.047) areas. Creatinine levels were also negatively associated with a thicker retina in the inner temporal (*P* = 0.002), inner superior (*P* = 0.026) and inner inferior (*P* < 0.001) areas. The HDLC levels were positively associated with thicker retinas in the inner nasal and inferior areas (both *P* = 0.029). There were no significant associations between the retinal thicknesses of the outer areas and blood data, except for creatinine levels and that in the outer superior area (*P* = 0.021) (Supplementary Table 2). Systemic data such as body height, body weight, body mass index (BMI), and systolic and diastolic blood pressure, as well as intraocular pressure showed no significant associations with retinal thicknesses except for retinal thickness of the outer temporal area and BMI (*P* = 0.039) (Supplementary Tables 3 and 4).

**Table 5:** Associations between retinal thickness and systemic factors.

ETDRS grid	Systemic Factors	Odds Ratio	95% Confidence Interval	*P* value
Center	HbA1c	0.547	0.341–0.878	0.012*
	Cre	0.809	0.159–4.127	0.799
	HDLC	1.062	0.890–1.267	0.478
Inner Temporal	HbA1c	0.632	0.407–0.983	0.042*
	Cre	0.410	0.005–0.323	0.002*
	HDLC	1.162	0.974–1.386	0.109
Inner Superior	HbA1c	0.722	0.471–1.106	0.134
	Cre	0.118	0.018–0.778	0.026*
	HDLC	1.174	0.984-1.400	0.083
Inner Nasal	HbA1c	0.771	0.508–1.171	0.222
	Cre	0.417	0.079–2.206	0.303
	HDLC	1.221	1.024–1.457	0.029*
Inner Inferior	HbA1c	0.635	0.406–0.994	0.047*
	Cre	0.027	0.003–0.226	< 0.001**
	HDLC	1.221	1.024–1.457	0.029*

Logistic regression analyses adjusted for age and sex. ETDRS grid, Early Treatment Diabetic Retinopathy Study grid; HbA1c, hemoglobin A1c; Cre, creatinine; HDLC, high-density lipoprotein cholesterol. Odds Ratios for HDLC were shown those per increase in 10 mg/dL. *P < 0.05, **P < 0.01.

## Discussion

The retinal thicknesses in each ETDRS grid obtained during the medical checkups showed that males had thicker retinas in the center and inner circles. The thicknesses in the ETDRS grid correlated with each other in most areas, and the retinal thicknesses between the superior and inferior areas of the inner and outer circles also correlated. The mean thickness of the superior area was greater than the inferior area in the inner and outer circles. However, some eyes had thicker inferior areas, which was observed more frequently in the inner circle of males. A thicker retina was associated with lower HbA1c levels in the center, and inner temporal and inferior areas; lower creatinine levels in the inner temporal, superior, and inferior areas; and higher HDLC levels in the inner nasal and inferior areas after adjusting for age and sex.

The retinal thicknesses in each ETDRS grid of the center 1-mm, and 4 quadrants of the inner (1–3 mm) and outer (3–6 mm) circles were mostly correlated with each other. These results were consistent with a previous report, which showed a similar correlation between the modified ETDRS grid of 1–3-mm and 3–9-mm circles in healthy eyes using wide-angle OCT^14^. The data confirmed that the studied eyes did not have ocular pathogeneses that affected local retinal thickness and/or ocular forms, such as glaucoma^15^ and posterior staphyloma^16^.

Males had thicker retinas on average, which is consistent with a previous report^14^. Other previous studies have revealed that photopic electroretinograms show greater amplitudes in females than in males in human^17^, and greater b-wave amplitudes in females than in males in Wistar rats^18^. Taken together, a thicker retina may not necessarily reflect better visual function, although a substantially thinner retina may reflect poorer visual function, as shown in geographic atrophy related to AMD^19^, and diabetic retinopathy^6,7^. The retina consists of retinal neurons, glial cells, vascular cells, microglial cells, and extracellular matrices (ECMs). It is known that components of ECMs are different between males and females in brain^20^ and peripheral nervous systems^21^ and expression of metalloproteases that may affect ECM volume are more produced in females than in males^22^. The volume of the other components than neural cells in the retina, such as ECMs, might be different between males and females.

Retinal thickness differences between the superior and inferior areas can be observed in glaucoma^15^ or highly myopic eyes^16^ in which local lesions develop. Although the current participants did not include those with diseased eyes and thicknesses between the superior and inferior areas of the ETDRS charts in both the inner and outer circles were correlated, the mean thickness was greater in the superior areas. The mean differences were 2.7 μm for males and 3.8 μm for females; males had 0.9%, and females had 1.2% thicker inner superior retina in average. Given that the difference in the retinal nerve fiber layer (RNFL) and ganglion cell layer (GCL) thickness between control and early glaucoma groups are 1–2 μm^15^, the impact of the original differences for the whole retinal thickness in the superior and inferior areas around the fovea should be considered when assessing the data.

Interestingly, 27.1% of the participants had thicker retinas in the inner inferior area, and the variation was observed more frequently in males. The retinal arteries are differently distributed in males and females, as indicated fundus photograph analyses, which was discussed as being the mechanism by which artificial intelligence identifies sex from fundus photographs^23^. Future research on differences in the distribution of retinal neurons between males and females is required.

Higher HbA1c levels were negatively associated with a thinner retina in the center area, where photoreceptors are the main component, after adjusting for age and sex. Patients with diabetes exhibit not only a thinner inner retinal layer, including RNFL and GCL^6^, but a shorter photoreceptor outer segment length and photoreceptor degeneration, which is correlated with choriocapillaris flow deficits^7^. These choriocapillaris flow deficits are more frequently observed in the patients who have a HbA1c level > 7%.^7^ High HbA1c levels are reportedly related to the thinning of the photoreceptor layer as well as total retinal thickness in the macular region in patients with diabetes^7,24^. Thus, HbA1c levels may be associated with photoreceptor degeneration. The association between higher HbA1c levels and inner temporal and inferior retinal thicknesses in the current study may also reflect the photoreceptor condition. In addition, HbA1c levels are related to the thinning of the RNFL in patients with diabetes^25,26^. Thus, both photoreceptors and nerve fibers may be affected in these areas. However, several reports have shown that HbA1c levels are positively correlated with RNFL thickness in patients with diabetes^27,28^. Given that HbA1c levels are positively correlated with diabetic hard exudates^29^, the increase in thickness reported in these studies may have involved exudative changes. In contrast, the current study excluded eyes with visible fundal changes, thus, the results may have reflected neuronal volume and retinal neurodegeneration.

The current study showed that higher creatinine levels were associated with thinner retinas in the inner temporal, superior, and inferior areas, where retinal nerve fibers and photoreceptors were abundant. This was consistent with a previous report that observed retinal thinning in patients with early stage non-diabetic and non-dialytic chronic kidney disease (CKD)^30^, and albumin to creatinine levels correlated with macular thinning in patients with prediabetes and diabetes^31^. Cystatin C, which is also associated with poor renal function, is associated with RNFL thinning^32^. Since a reduction in retinal perfusion has only been observed in the advanced CKD stages, they speculated the neurodegenerative mechanism of accumulated toxic materials due to early CKD^30^. Increases in creatinine levels also affect photoreceptors; as the levels increase, disruptions of the external limiting membrane and ellipsoid zone are observed in OCT images^33^.

Serum HDLC levels were positively associated with thicker retinas in the inner nasal and inferior areas. As circulating HDLC concentrations are inversely correlated with the risk of cardio-metabolic disorders^34^, higher HDLC levels may be related to healthy conditions. In this regard, our results may suggest that lower HDLC levels may be related to neurodegeneration. However, the interpretation of HDLC levels in healthy or pathological conditions may not be simple. Although higher HDLC levels were positively correlated with RNFL thickness in a large population-based study^32^, they were also negatively correlated in patients with diabetes^27^. Another study showed that HDLC levels were negatively correlated with RNFL thickness as a whole; however, the same study showed that HDLC levels were positively correlated in females if the HDLC levels were moderate^35^. HDLC is necessary for the cholesterol efflux system to regulate cellular lipid metabolism, and both the levels and functionality of HDLC are important for metabolism^34,36^. Decreased circulating concentrations of sphingosine-1-phosphate within HDLC particles can impair the protective effect of HDLC, at least in part, on atherosclerosis^34^. The efflux of lipids originating from the phagocytosed photoreceptor outer segment in the retinal pigment epithelium (RPE) is regulated by HDLC^37^. HDLC mimetics can protect the RPE and photoreceptors in mice^38^. Thus, HDLC metabolism may also affect photoreceptors and RPE conditions.

This study had limitations. It was retrospective, and the participants were those who attended the medical checkup; therefore, those who had systemic diseases with or without treatment, as well as healthy people were included. However, eyes with retinal lesions and glaucoma, were excluded based on the OCT images and fundus photographs. Visual acuity was measured, but without best-correction, and was not assessed in the study. Measurement of axial length was not included in the test items of medical checkup, and not adjusted in measuring mean retinal thickness in OCT images. However, highly myopic eyes were excluded in the study, and the influence may have been minimal. The nationalities of the participants were not uniform; although, all participants were from Asian countries. This was not a case-control study; all eyes could be assumed to have no ocular diseases, which enabled us to analyze the potential effects of background factors on the retina.

The study showed that retinal thickness varied according to sex and systemic data; the serum levels of HbA1c, creatinine, and HDLC. The differences between the superior and inferior areas also varied, particularly in males; which were evident within 3 mm circle areas around the fovea. These results indicate that retinal thickness may be influenced by environmental factors such as dietary habits and genetic background, which potentially affect blood test results in addition to retinal diseases. The researchers may pay attention to the systemic conditions, such as glucose and lipid metabolisms and renal conditions, when assessing the retinal thickness, while we usually see only ocular data in clinical studies. Further studies with longitudinal observation with or without systemic treatments are warranted.

## Methods

### Participants

The participants were Japanese or visitors from other Asian countries who underwent a medical checkup at Fujita Medical Innovation Center, Tokyo, Japan, from October 2023 to September 2024. Those with eye diseases based on OCT images and fundus photographs such as AMD, epiretinal membrane, diabetic retinopathy, high myopia with or without posterior staphyloma, macular dystrophy, or retinitis pigmentosa, and those with glaucomatous retinal findings were excluded. Eyes without clear images were also excluded. The right eyes of the participants were evaluated.

This study adhered to the principles of the Declaration of Helsinki and was approved by the Fujita Health University Ethics Committee (approval number: HM23-276). Written informed consent was obtained from all the participants to use their data for research purposes.

### Eye examinations

A spectral-domain OCT and color fundus camera system, the Maestro2 (Topcon Corporation, Tokyo, Japan), was used to record retinal sectional images (5-line scan), retinal 3-dimensional images (3D wide scan), and fundus photographs. Retinal thickness was measured using 3-dimensional OCT images with built-in device software, according to the ETDRS grid of the retina in 6 mm diameter areas. Intraocular pressure was measured using a pneumatic tonometer, the CT-1P (Topcon Corporation).

### Blood sample examinations

Blood samples were collected at the same day as the eye examinations. The Hb A1c, creatinine, TG, TC, HDLC, and LDLC serum levels were analyzed.

### Systemic data measurements

Body height and weight were measured to calculate body mass index, and systolic and diastolic blood pressures were also measured during the medical checkup.

### Statistical analyses

IBM SPSS Statistics (version 29.0; IBM Corp., Armonk, NY, USA) was used for all the statistical analyses. The Mann–Whitney U test, Pearson’s correlation coefficient analysis, paired t-test, chi-square test, and logistic regression analyses were used. The eyes with retinal thickness greater than the median value were placed in the thicker group in each ETDRS grid area. The eyes of the participants whose age greater than the median value were placed in the older group in each ETDRS grid area. Statistical significance was set at *P* < 0.05. All data are presented as the mean ± standard deviation values.

## Electronic supplementary material

Below is the link to the electronic supplementary material.

Supplementary Material 1

## Data Availability

The datasets generated during and/or analyzed during the current study are available from the corresponding author on reasonable request.

## References

[1] Heier, J. S. et al. Pegcetacoplan for the treatment of geographic atrophy secondary to age-related macular degeneration (OAKS and DERBY): two multicentre, randomised, double-masked, sham-controlled, phase 3 trials. *Lancet* **402**, 1434–1448. 10.1016/S0140-6736(23)01520-9 (2023). 37865470 10.1016/S0140-6736(23)01520-9

[2] Khanani, A. M. et al. Efficacy and safety of Avacincaptad Pegol in patients with geographic atrophy (GATHER2): 12-month results from a randomised, double-masked, phase 3 trial. *Lancet* **402**, 1449–1458. 10.1016/S0140-6736(23)01583-0 (2023). 37696275 10.1016/S0140-6736(23)01583-0

[3] Nagai, N. et al. Extent of complete retinal pigment epithelial and outer retinal atrophy with foveal center involvement is associated with visual acuity. *Ophthalmol. Sci.* **5**, 100612. 10.1016/j.xops.2024.100612 (2025). 39963552 10.1016/j.xops.2024.100612 PMC11832003

[4] Medeiros, F. A. et al. A combined index of structure and function for staging glaucomatous damage. *Arch. Ophthalmol.* **130**, 1107–1116. 10.1001/archophthalmol.2012.827 (2012). 23130365 10.1001/archophthalmol.2012.827 PMC3787828

[5] Aizawa, N. et al. Preperimetric Glaucoma prospective observational study (PPGPS): design, baseline characteristics, and therapeutic effect of Tafluprost in preperimetric glaucoma eye. *PLoS One*. **12**, e0188692. 10.1371/journal.pone.0188692 (2017). 29236784 10.1371/journal.pone.0188692 PMC5728557

[6] Nagai, N., Mushiga, Y. & Ozawa, Y. Evaluating fine changes in visual function of diabetic eyes using spatial-sweep steady-state pattern electroretinography. *Sci. Rep.* **13**, 13686. 10.1038/s41598-023-40686-5 (2023). 37608045 10.1038/s41598-023-40686-5 PMC10444753

[7] Nagai, N., Mushiga, Y. & Ozawa, Y. Diabetic choriocapillaris flow deficits affect the outer retina and are related to hemoglobin A1c and systolic blood pressure levels. *Sci. Rep.* **13**, 22570. 10.1038/s41598-023-50132-1 (2023). 38114663 10.1038/s41598-023-50132-1 PMC10730885

[8] Ueta, T. et al. Development of typical age-related macular degeneration and polypoidal choroidal vasculopathy in fellow eyes of Japanese patients with exudative age-related macular degeneration. *Am. J. Ophthalmol.* **146**, 96–101. 10.1016/j.ajo.2008.03.002 (2008). 18439567 10.1016/j.ajo.2008.03.002

[9] Zarranz-Ventura, J. et al. The neovascular age-related macular degeneration database: report 2: incidence, management, and visual outcomes of second treated eyes. *Ophthalmology* **121**, 1966–1975. 10.1016/j.ophtha.2014.04.026 (2014). 24953791 10.1016/j.ophtha.2014.04.026

[10] Zarranz-Ventura, J. et al. Characterization of punctate inner choroidopathy using enhanced depth imaging optical coherence tomography. *Ophthalmology* **121**, 1790–1797. 10.1016/j.ophtha.2014.03.011 (2014). 24856311 10.1016/j.ophtha.2014.03.011

[11] Nagai, N. et al. Macular pigment optical density and photoreceptor outer segment length as predisease biomarkers for Age-Related macular degeneration. *J. Clin. Med.* **9**10.3390/jcm9051347 (2020). 10.3390/jcm9051347 PMC7290696 32380638

[12] Harada, N., Nagai, N., Mushiga, Y. & Ozawa, Y. Choriocapillaris flow imbalance in fellow eyes in Age-Related macular degeneration. *Invest. Ophthalmol. Vis. Sci.* **63**, 13. 10.1167/iovs.63.9.13 (2022). 35943731 10.1167/iovs.63.9.13 PMC9379328

[13] Nagai, N., Mushiga, Y. & Ozawa, Y. Retinal pigment epithelial abnormality and choroidal large vascular flow imbalance are associated with choriocapillaris flow deficits in Age-Related macular degeneration in fellow eyes. *J. Clin. Med.* **12**10.3390/jcm12041360 (2023). 10.3390/jcm12041360 PMC9965486 36835897

[14] Kicinski, K. & Gawecki, M. Choroidal and retinal thicknesses in healthy eyes measured with Ultra-Wide-Field optical coherence tomography. *Diagnostics (Basel)*. **14**10.3390/diagnostics14111114 (2024). 10.3390/diagnostics14111114 PMC11171910 38893640

[15] Lee, S. Y., Lee, E. K., Park, K. H., Kim, D. M. & Jeoung, J. W. Asymmetry analysis of macular inner retinal layers for Glaucoma diagnosis: Swept-Source optical coherence tomography study. *PLoS One*. **11**, e0164866. 10.1371/journal.pone.0164866 (2016). 27764166 10.1371/journal.pone.0164866 PMC5072638

[16] Deng, J. et al. Increased vertical asymmetry of macular retinal layers in myopic Chinese children. *Curr. Eye Res.* **44**, 225–235. 10.1080/02713683.2018.1530360 (2019). 30335521 10.1080/02713683.2018.1530360

[17] Brule, J., Lavoie, M. P., Casanova, C., Lachapelle, P. & Hebert, M. Evidence of a possible impact of the menstrual cycle on the reproducibility of scotopic ERGs in women. *Doc. Ophthalmol.* **114**, 125–134. 10.1007/s10633-007-9045-1 (2007). 17273847 10.1007/s10633-007-9045-1

[18] Tyszkiewicz, C. et al. Sex-related differences in retinal function in Wistar rats: implications for toxicity and safety studies. *Front. Toxicol.* **5**, 1176665. 10.3389/ftox.2023.1176665 (2023). 37313214 10.3389/ftox.2023.1176665 PMC10259507

[19] Ozawa, Y. et al. Effects of epigenetic modification of PGC-1alpha by a chemical chaperon on mitochondria biogenesis and visual function in retinitis pigmentosa. *Cells* **11**10.3390/cells11091497 (2022). 10.3390/cells11091497 PMC9099608 35563803

[20] Batzdorf, C. S. et al. Sexual dimorphism in extracellular matrix composition and viscoelasticity of the healthy and inflamed mouse brain. *Biology (Basel)*. **11**10.3390/biology11020230 (2022). 10.3390/biology11020230 PMC8869215 35205095

[21] Mecklenburg, J. et al. Transcriptomic sex differences in sensory neuronal populations of mice. *Sci. Rep.* **10**, 15278. 10.1038/s41598-020-72285-z (2020). 32943709 10.1038/s41598-020-72285-z PMC7499251

[22] Simon, L. R., Scott, A. J., Rios, F., Zembles, L., Masters, K. S. & J. & Cellular-scale sex differences in extracellular matrix remodeling by valvular interstitial cells. *Heart Vessels*. **38**, 122–130. 10.1007/s00380-022-02164-2 (2023). 36070095 10.1007/s00380-022-02164-2 PMC10120251

[23] Yamashita, T. et al. Factors in color fundus photographs that can be used by humans to determine sex of individuals. *Transl Vis. Sci. Technol.* **9** (4). 10.1167/tvst.9.2.4 (2020). 10.1167/tvst.9.2.4 PMC7255626 32518709

[24] Chua, S. Y. L. et al. Associations between HbA1c across the normal range, diagnosed, and undiagnosed diabetes and retinal layer thickness in UK biobank cohort. *Transl Vis. Sci. Technol.* **12**, 25. 10.1167/tvst.12.2.25 (2023). 36795065 10.1167/tvst.12.2.25 PMC9940769

[25] Toprak, I., Fenkci, S. M., Yaylali, F., Martin, G., Yaylali, V. & C. & Early retinal neurodegeneration in preclinical diabetic retinopathy: a multifactorial investigation. *Eye (Lond)*. **34**, 1100–1107. 10.1038/s41433-019-0646-1 (2020). 31654034 10.1038/s41433-019-0646-1 PMC7253424

[26] Shi, R. et al. Alterations in retinal nerve fiber layer thickness in early stages of diabetic retinopathy and potential risk factors. *Curr. Eye Res.* **43**, 244–253. 10.1080/02713683.2017.1387669 (2018). 29111833 10.1080/02713683.2017.1387669

[27] Wu, J. et al. Dyslipidemia and reduced retinal layer thicknesses in mild to moderate non-proliferative diabetic retinopathy. *Am. J. Transl Res.* **16**, 5718–5727. 10.62347/EHTP6496 (2024). 39544759 10.62347/EHTP6496 PMC11558433

[28] Mititelu, M. et al. Retinal thickness and morphology changes on OCT in youth with type 2 diabetes: findings from the TODAY study. *Ophthalmol. Sci.* **2**, 100191. 10.1016/j.xops.2022.100191 (2022). 36531589 10.1016/j.xops.2022.100191 PMC9754955

[29] Hiran, H. M. et al. Association of serum lipid profile and other systemic risk factors with retinal hard exudates in diabetic retinopathy. *Int. Ophthalmol.* **44**, 338. 10.1007/s10792-024-03263-x (2024). 39095678 10.1007/s10792-024-03263-x PMC11297163

[30] Zeng, X. et al. Retinal neurovascular impairment in Non-diabetic and Non-dialytic chronic kidney disease patients. *Front. Neurosci.* **15**, 703898. 10.3389/fnins.2021.703898 (2021). 34867144 10.3389/fnins.2021.703898 PMC8639216

[31] Kirthi, V. et al. Associations between dysglycemia, retinal neurodegeneration, and microalbuminuria in prediabetes and type 2 diabetes. *Retina* **42**, 442–449. 10.1097/IAE.0000000000003337 (2022). 35188489 10.1097/IAE.0000000000003337

[32] Rauscher, F. G. et al. Renal function and lipid metabolism are major predictors of circumpapillary retinal nerve fiber layer thickness-the LIFE-Adult study. *BMC Med.* **19**, 202. 10.1186/s12916-021-02064-8 (2021). 34488766 10.1186/s12916-021-02064-8 PMC8422631

[33] Saxena, S. et al. Increased serum levels of Urea and creatinine are surrogate markers for disruption of retinal photoreceptor external limiting membrane and inner segment ellipsoid zone in type 2 diabetes mellitus. *Retina* **37**, 344–349. 10.1097/IAE.0000000000001163 (2017). 28118284 10.1097/IAE.0000000000001163

[34] Kang, H., Song, J. & Cheng, Y. HDL regulates the risk of cardiometabolic and inflammatory-related diseases: focusing on cholesterol efflux capacity. *Int. Immunopharmacol.* **138**, 112622. 10.1016/j.intimp.2024.112622 (2024). 38971111 10.1016/j.intimp.2024.112622

[35] Ma, Y. et al. Association of retinal nerve Fiber layer thinning with elevated high density lipoprotein cholesterol in UK biobank. *Invest. Ophthalmol. Vis. Sci.* **65**10.1167/iovs.65.11.12 (2024). 10.1167/iovs.65.11.12 PMC11382965 39240552

[36] Kim, Y. K., Hong, H. K., Yoo, H. S., Park, S. P. & Park, K. H. AICAR upregulates ABCA1/ABCG1 expression in the retinal pigment epithelium and reduces bruch’s membrane lipid deposit in ApoE deficient mice. *Exp. Eye Res.* **213**, 108854. 10.1016/j.exer.2021.108854 (2021). 34808137 10.1016/j.exer.2021.108854

[37] Ishida, B. Y., Duncan, K. G., Bailey, K. R., Kane, J. P. & Schwartz, D. M. High density lipoprotein mediated lipid efflux from retinal pigment epithelial cells in culture. *Br. J. Ophthalmol.* **90**, 616–620. 10.1136/bjo.2005.085076 (2006). 16622093 10.1136/bjo.2005.085076 PMC1857047

[38] Su, F. et al. A novel HDL-Mimetic peptide HM-10/10 protects RPE and photoreceptors in murine models of retinal degeneration. *Int. J. Mol. Sci.* **20**10.3390/ijms20194807 (2019). 10.3390/ijms20194807 PMC6801888 31569695

