# Supplementary material for: Associations between retinal thickness and background factors in eyes without retinal diseases
Source: Sci Rep. 2025 Jul 1;15:21796. doi: 10.1038/s41598-025-06863-4 (PMC12215744; doi:10.1038/s41598-025-06863-4)
Supplement: Supplementary file 1 — Supplementary Material 1 [file 41598_2025_6863_MOESM1_ESM.pdf]

Associations Between Retinal Thickness and Background Factors in Eyes without Retinal Diseases

Yoko Ozawa M.D., Ph.D.[1,2], Noriko Onozato[1], Haruna Togawa[1], Shigeto Shimmura M.D., Ph.D.[1,2]

[1]Department of Clinical Regenerative Medicine, Fujita Medical Innovation Center Tokyo, Eye Center, Fujita Health University Haneda Clinic

[2]Department of Ophthalmology, Keio University School of Medicine

*Correspondence author

Yoko Ozawa M.D., Ph.D.

Professor

Department of Clinical Regenerative Medicine

Fujita Medical Innovation Center Tokyo

Eye Center, Fujita Health University, Haneda Clinic

ozawa.a5@keio.jp and yoko.ozawa@fujita-hu.ac.jp

ORCID: 0000-0003-4797-5705

Supplementary Table 1. Differences in retinal thickness between age below 49 y/o or over

Retina thickness	Age < 49 y/o (n=138)	Age ≥ 49 y/o (n=128)	P value
Center (μm)	236.6 ± 19.2	239.0 ± 21.4	0.247
Inner Temporal (μm)	306.1 ± 14.0	304.7 ± 14.9	0.597
Inner Superior (μm)	319.8 ± 13.5	316.7 ± 15.5	0.168
Inner Nasal (μm)	318.0 ± 14.4	315.5 ± 15.9	0.405
Inner Inferior (μm)	316.8 ± 13.8	313.5 ± 14.9	0.173
Outer Temporal (μm)	265.3 ± 11.9	264.4 ± 12.9	0.446
Outer Superior (μm)	277.4 ± 11.7	274.6 ± 13.5	0.049*
Outer Nasal (μm)	294.0 ± 13.7	292.0 ± 16.2	0.365
Outer Inferior (μm)	263.8 ± 11.7	261.7± 14.5	0.176

Data are presented as mean ± standard deviation. Retinal thicknesses were analyzed using the Early Treatment Diabetic Retinopathy Study grid. Mann-Whitney U test. *P<0.05.

Supplementary Table 2. Associations between retinal thickness (outer circle) and systemic factors

ETDRS grid	Systemic Factors	Odds Ratio	95% Confidence Interval	P value
Outer Temporal	HbA1c	0.924	0.616-1.386	0.702
	Cre	0.829	0.165-4.176	0.821
	HDLC	1.030	0.864-1.229	0.772
Outer Superior	HbA1c	0.761	0.495-1.171	0.215
	Cre	0.097	0.014-0.699	0.021*
	HDLC	1.062	0.890-1.267	0.499
Outer Nasal	HbA1c	1.150	0.763-1.733	0.506
	Cre	0.282	0.050-1.596	0.152
	HDLC	1.051	0.8818-1.254	0.539
Outer Inferior	HbA1c	0.923	0.615-1.386	0.700
	Cre	0.537	0.103-2.795	0.460
	HDLC	0.980	0.822-1.169	0.813

Logistic regression analyses adjusted for age and sex. ETDRS grid, Early Treatment Diabetic Retinopathy Study grid; HbA1c, hemoglobin A1c; Cre, creatinine; HDLC, high-density lipoprotein cholesterol. Odds Ratios for HDLC were shown those per increase in 10 mg/dL. *P<0.05.

Supplementary Table 3. Associations between retinal thickness and other systemic factors

ETDRS grid	Systemic Factors	Odds Ratio	95% Confidence Interval	P value
Center	Body Height	1.028	0.983-1.076	0.225
	Body Weight	0.986	0.986-1.036	0.388
	Body Mass Index	1.016	0.939-1.098	0.694
	Systolic Blood Pressure	1.001	0.997-1.006	0.556
	Diastolic Blood Pressure	0.995	0.974-1.017	0.653
Inner Temporal	Body Height	0.999	0.955-1.045	0.961
	Body Weight	0.982	0.958-1.007	0.154
	Body Mass Index	0.942	0.869-1.021	0.147
	Systolic Blood Pressure	1.001	0.996-1.005	0.728
	Diastolic Blood Pressure	0.990	0.969-1.012	0.367
Inner Superior	Body Height	0.996	0.952-1.041	0.857
	Body Weight	0.983	0.959-1.008	0.180
	Body Mass Index	0.953	0.881-1.031	0.229
	Systolic Blood Pressure	1.002	0.996-1.008	0.519
	Diastolic Blood Pressure	0.999	0.978-1.021	0.953
Inner Nasal	Body Height	0.996	0.952-1.041	0.858
	Body Weight	1.004	0.979-1.029	0.758
	Body Mass Index	1.020	0.943-1.103	0.620
	Systolic Blood Pressure	1.001	0.996-1.006	0.606
	Diastolic Blood Pressure	0.996	0.975-1.017	0.702
Inner Inferior	Body Height	1.009	0.964-1.055	0.710
	Body Weight	0.985	0.961-1.010	0.246
	Body Mass Index	0.946	0.874-1.024	0.167
	Systolic Blood Pressure	1.001	0.997-1.005	0.683
	Diastolic Blood Pressure	0.984	0.963-1.005	0.186
Outer Temporal	Body Height	-0.002	0.954-1.043	0.913
	Body Weight	-0.024	0.952-1.001	0.058
	Body Mass Index	-0.084	0.849-0.996	0.039*
	Systolic Blood Pressure	-0.004	0.965-1.006	0.432
	Diastolic Blood Pressure	-0.012	0.967-1.009	0.265
Outer Superior	Body Height	0.998	0.936-1.023	0.335
	Body Weight	0.976	0.954-1.002	0.075

	Body Mass Index	0.919	0.868-1.016	0.117
	Systolic Blood Pressure	0.996	0.981-1.008	0.394
	Diastolic Blood Pressure	0.988	0.982-1.024	0.824
Outer Nasal	Body Height	0.978	0.939-1.026	0.410
	Body Weight	1.004	0.960-1.008	0.196
	Body Mass Index	0.960	0.889-1.038	0.307
	Systolic Blood Pressure	0.997	0.988-1.005	0.449
	Diastolic Blood Pressure	1.000	0.979-1.021	0.998
Outer Inferior	Body Height	0.982	0.947-1.035	0.659
	Body Weight	0.991	0.979-1.015	0.460
	Body Mass Index	0.978	0.905-1.056	0.566
	Systolic Blood Pressure	0.994	0.981-1.008	0.388
	Diastolic Blood Pressure	0.986	0.965-1.007	0.193

Logistic regression analyses adjusted for age and sex. ETDRS grid, Early Treatment Diabetic Retinopathy Study grid. *P<0.05.

Supplementary Table 4. Associations between retinal thickness and intraocular pressure

ETDRS grid	Odds Ratio	95% Confidence Interval	P value
Center	0.997	0.905-1.099	0.957
Inner Temporal	1.007	0.913-1.110	1.007
Inner Superior	1.048	0.951-1.115	0.341
Inner Nasal	1.013	0.919-1.116	0.800
Inner Inferior	0.966	0.877-1.065	0.488
Outer Temporal	0.986	0.896-1.085	0.775
Outer Superior	1.041	0.946-1.146	0.407
Outer Nasal	1.036	0.941-1.140	0.468
Outer Inferior	0.981	0.892-1.080	0.699

Logistic regression analyses adjusted for age and sex. ETDRS grid, Early Treatment Diabetic Retinopathy Study grid.
